# Essen Risk Score in Prediction of Myocardial Infarction After Transient Ischemic Attack or Ischemic Stroke Without Prior Coronary Artery Disease

**DOI:** 10.1161/STROKEAHA.119.025831

**Published:** 2019-10-22

**Authors:** Marion Boulanger, Linxin Li, Shane Lyons, Nicola G. Lovett, Magdalena M. Kubiak, Louise Silver, Emmanuel Touzé, Peter M. Rothwell

**Affiliations:** 1 From the Centre for Prevention of Stroke and Dementia, Nuffield Department of Clinical Neurosciences, John Radcliffe Hospital, University of Oxford, United Kingdom (M.B., L.L., S.L., N.G.L., M.M.K., L.S., P.M.R.); 2 Normandie Université, UNICAEN, CHU Caen Normandie, Service de Neurologie, INSERM U1237, avenue de la Côte de Nacre, Caen, France (M.B., E.T.).

**Keywords:** coronary artery disease, myocardial infarction, risk, secondary prevention, transient ischemic attack

## Abstract

More intensive secondary prevention with newer drugs may be cost-effective in patients with coronary artery disease (CAD). Whether some subgroups of patients who had a transient ischemic attack (TIA) or ischemic stroke, but no prior CAD are at similar high risk of myocardial infarction as those with prior CAD remains unclear. We determined whether the Essen score identified a subset of TIA/stroke patients without known prior CAD who, nevertheless, had a high risk of myocardial infarction on current secondary prevention management.

**Methods—:**

In a population-based cohort (Oxford Vascular Study) of consecutive TIA or ischemic stroke patients recruited from 2002 to 2014, 10-year actuarial risks of myocardial infarction and of recurrent ischemic stroke were determined by face-to-face follow-up in patients with and without prior CAD using Kaplan-Meier analyses. Predictive value of the Essen score was assessed with C statistic.

**Results—:**

Of 2555 patients with TIA/stroke (13 070 patient-years of follow-up), 10-year risk of myocardial infarction in those without prior CAD (n=2017, 78.9%) ranged from 0.9% (95% CI, 0–1.9) at Essen score ≤1 to 29.8% (95% CI, 7.7–46.6) in those with a score ≥5 (C statistic =0.64 [95% CI, 0.57–0.71]; *P*<0.001). The score tended to be less predictive (difference: *P*=0.0460) for the risk of recurrent ischemic stroke (C statistic =0.57 [95% CI, 0.54–0.60]). Compared with patients with prior CAD (n=538, 21.1%), an Essen risk score of ≥4 (n=294, 11.5%) in those without prior CAD identified a subgroup at similar high 10-year risks of myocardial infarction (17.2% [95% CI, 6.9–26.3] versus 16.9% [95% CI, 11.5–22.0]) and of recurrent stroke (40.4% [95% CI, 26.7–51.6] versus 32.4% [95% CI, 25.2–38.8]).

**Conclusions—:**

The Essen score is a simple clinical score to risk-stratify patients with TIA/stroke without prior CAD and to identify subsets who may be at sufficiently high risk of myocardial infarction and recurrent stroke to justify more intensive treatment or inclusion in trials.

## Abstract 2

Supplemental Digital Content is available in the text.

**See related article, p 3335**

The long-term risk of myocardial infarction (MI) and recurrent ischemic stroke after a transient ischemic attack (TIA) or ischemic stroke is particularly high in patients with prior coronary artery disease (CAD).^1,2^ Recent trials of more intensive secondary prevention, such as those of lipid-lowering with the PCSK9 (proprotein convertase subtilisin-kexin type 9) inhibitors^3–5^ or those combining antithrombotic treatments,^6^ focused on patients with prior CAD. However, whether some subgroups of patients with TIA/stroke without prior CAD have a similar high long-term risk of MI or recurrent ischemic stroke remains uncertain. Although the SPARCL trial^7^ showed that higher dose of statin should be standard of care in patients with TIA/stroke, it is uncertain whether more intensive treatment might be justified in certain subgroups.

Long-term follow-up of patients with TIA/stroke has shown that the risks of death due to recurrent stroke and death due to coronary events are similar.^1^ However, risk stratification schemes tend to focus on risk of recurrent stroke.^8–13^ Nevertheless, since risk scores to predict the risk of recurrent ischemic stroke are mostly based on risk factors for, or clinical manifestations of, atherosclerosis they may also predict coronary events. Therefore, rather than attempting to derive and validate a new score to predict coronary events after TIA/stroke, we determined the predictive value of the Essen stroke risk score for risk of MI after TIA/stroke. The Essen risk score is a simple clinical score that was derived to predict the 1-year risk of recurrent ischemic stroke after ischemic stroke based on the presence of prior vascular comorbidities, including current smoking, previous TIA or ischemic stroke, prior CAD, peripheral artery disease, hypertension, diabetes mellitus, and age and is one of the most established scores for patients with stroke.^11,13^

The Essen score has been validated previously, but further validation would be helpful for several reasons. First, previous validations have looked only at short-term risk, predicting the 1-year risk of recurrent stroke,^11,13–19^ or the 1-year risk of the combined outcome of recurrent stroke, MI, and cardiovascular death^14,17,19^ or the 1-year risk of combined outcome of recurrent stroke or cardiovascular death.^18^ Second, the score was derived on patients recruited into a trial cohort in the early 1990s, and the validation cohorts involved patients recruited in trials or hospital-based registries, mostly in the early 2000s before the widespread use of higher-dose statins.^7^ The absolute risk of MI after TIA/stroke has decreased over the past few decades,^1^ and there are relatively few recent data on the overall residual vascular risk in patients with TIA/stroke on current guideline-based management, particularly on long-term follow-up. Third, previous validation studies have focused mainly on patients with stroke due to large artery disease, have excluded patients with TIA, and have suggested that the score might perform less well in those with stroke due to small vessel disease.^19^ Finally, no study has looked at whether the score predicts risk of MI as well as stroke, and none have assessed predictive value in patients with no prior CAD.

We aimed to determine whether the Essen score identified a subset of patients with TIA/stroke without known prior CAD who nevertheless had a high risk of MI on current secondary prevention management and might, therefore, benefit from more intensive treatment or might be included in future trials.

## Methods

Requests for anonymized data will be considered by the corresponding author.

The OXVASC (Oxford Vascular Study) is an ongoing population-based study of the incidence and outcome of all acute vascular events in 92 728 individuals, irrespective of age, registered with 100 family physicians in 9 general practices in Oxfordshire, United Kingdom.^20^ The analysis reported here included consecutive cases, irrespective of age, with a TIA or ischemic stroke from 2002 to 2014, with follow-up until 2016. The OXVASC study design has been described in detail previously.^20^ Briefly, multiple overlapping methods of hot and cold pursuit were used to achieve near-complete ascertainment of all individuals with TIA or stroke.^21,22^ These include (1) a daily, rapid access TIA and stroke clinic to which participating general practitioners and the local emergency department refer individuals with suspected TIA or minor stroke; (2) daily searches of admissions to the medical, stroke, neurology, and other relevant wards; (3) daily searches of the local emergency department attendance register; (4) daily searches of in-hospital death records via the Bereavement Office; (5) monthly searches of all death certificates and coroner’s reports for out-of-hospital deaths; (6) monthly searches of general practitioner diagnostic coding and hospital discharge codes; and (7) monthly searches of all brain and vascular imaging referrals.

Demographic data, prior comorbidities, and medication were collected from face-to-face interviews and cross-referenced with primary care records. All cases were investigated and treated according to current guidelines. Patients were classified as having a prior CAD if they had at least one of the following: previous MI; unstable angina; angina; previous percutaneous coronary intervention; or coronary artery bypass graft surgery. Written informed consent or assent from relatives was obtained in all patients. OXVASC was approved by our local research ethics committee.

Standard World Health Organization definitions were used for TIA and stroke, which have been reported previously.^20,21^ All cases were investigated according to current guidelines and reviewed by a senior neurologist with stroke expertise (P.M. Rothwell) to ensure consistency over time and TIA/stroke cause was categorized according to the modified TOAST (Trial of ORG 10172 in Acute Stroke Treatment) criteria (Table I in the online-only Data Supplement).^23^ Differentiation between TIA and stroke was based on the WHO 24-hour cutoff for symptom duration (ie, events were classified as TIA even if diffusion-weighted imaging was positive if symptoms resolved within 24 hours), and we have kept the same definition over time. Patients were prescribed standard secondary prevention medication according to contemporary guidelines, including antiplatelet or anticoagulant treatment, as appropriate, blood pressure-lowering medication and statin treatment to achieve a target of blood pressure <140/90 and LDL (low-density lipoprotein) <2.6 mmol/L, both at baseline and at the time of study follow-up visits.

All patients were followed up face-to-face at 1, 6, 12, 60, and 120 months by a study nurse or physician and subsequent vascular events identified and risk factor control evaluated and treatment revised as necessary. For patients who had moved out of the study area, telephone follow-up was done, and patients with dementia were followed up via a carer or by assessment in a nursing home. All MIs that presented to medical attention would also be identified by ongoing daily case-ascertainment of all acute vascular events in the study population. All study patients were also notified to the UK Office of National Statistics such that all deaths were reported back to the study with causes. Deaths were also identified by regular review of primary care records and by regular contact with the Coroner’s Office to ascertain out-of-hospital deaths.

MI was defined using standard criteria and remained unchanged over the study period.^24^ Recurrent ischemic stroke was defined as new neurological deficit fitting the definition for ischemic stroke occurring after a period of neurological stability or improvement.

### Statistical Analysis

We determined the 10-year actuarial risk of MI and of recurrent ischemic stroke from Kaplan-Meier analyses in patients with TIA/stroke with and without prior CAD. Among the latter subgroup, we stratified risk by the Essen score but excluded the history of other cardiac disease (ie, cardiac disease other than MI or atrial fibrillation; Table 1) because this variable was not fully defined in previous reports.^11,14,15^ Essen scores in patients without prior CAD, therefore, ranged from 0 to 7: 1 point is attributed for age 65 to 75 years; 2 for age >75 years and 1 for each history of prior hypertension, diabetes mellitus, peripheral artery disease, current smoking, or previous TIA or ischemic stroke. A small number of patients with missing value on the Essen score were excluded from the analyses. Discrimination (ie, how well the score distinguished between people with the outcome and those without) was assessed with the C statistic. Difference in the discrimination of the score for the risk of MI and for the risk of recurrent ischemic stroke was assessed by a Z-test. Age- and sex-adjusted risk associations between variables of the Essen score and MI were determined by Cox regression. In additional analyses in patients without prior CAD, we determined the 10-year risk of MI or recurrent ischemic stroke based on the Essen score and stratified by presence of large artery disease (TOAST) subtype, which have been shown to carry one of the highest risks of MI and recurrent ischemic stroke,^2^ and difference was assessed by log-rank *P*. Also, we determined the predictive value of adding the variable presence of large artery disease (TOAST) subtype as a categorical variable to the Essen score for the risk of MI and of recurrent ischemic stroke, with 1 point being attributed for the presence of large artery disease subtype and 0 point for any other subtypes than large artery disease. All analyses were done using R version 3.1.3.

**Table 1.**
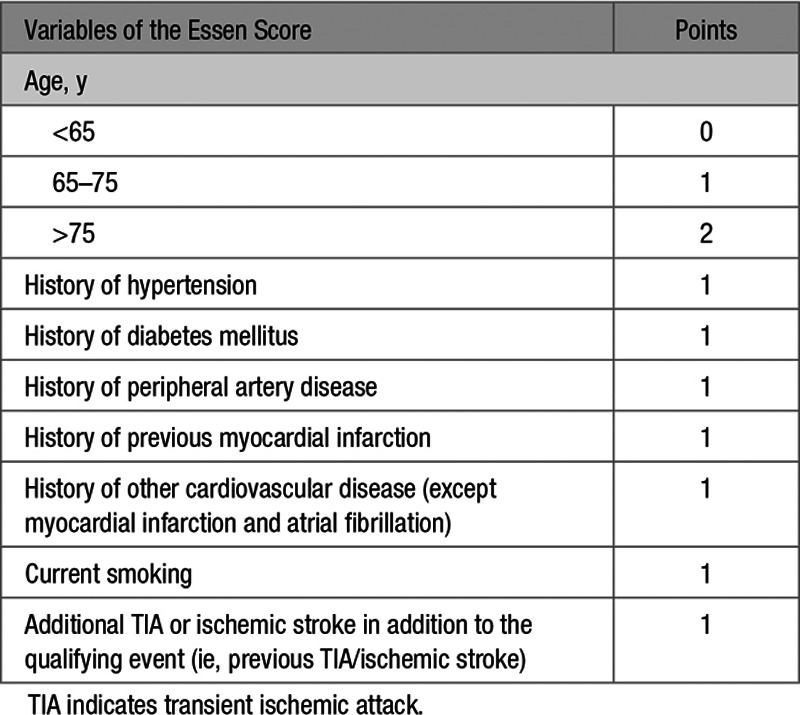
The Essen Risk Score^11,13^

## Results

Of 2555 patients with TIA/stroke, those without prior CAD (n=2017; 78.9%) were younger, had less prior vascular comorbidities, except for current smoking, and were less likely to be on antithrombotics, antihypertensives and statins before the TIA/stroke than those with prior CAD (*P*<0.001, Table 2). Rates of secondary preventive therapies use were high at 1-year follow-up, but rates of antithrombotics and antihypertensives use remained lower in patients without CAD than in those with (Table 2). The prevalence of TIA/stroke due to large artery disease was similar in patients with and without prior CAD, but TIA/stroke was less often due to cardioembolism in patients without CAD (Table 2).

**Table 2.**
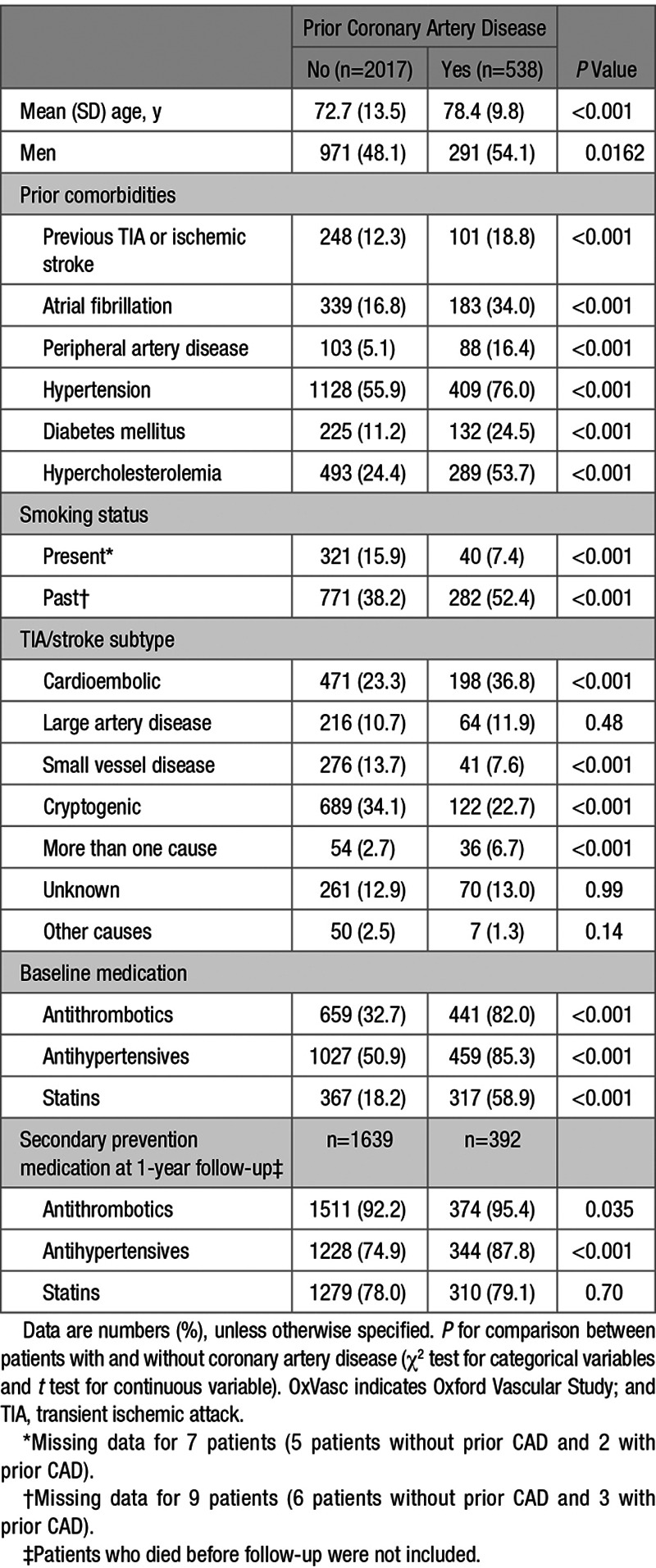
Baseline Characteristics of OxVasc Patients With TIA or Stroke Included in the Analysis and Medication at Baseline and on Follow-Up

During 13 070 patient-years of follow-up, there were 118 MIs after a median (range) of 5.6 (0–14.6) years, of which 70 (59.3%) occurred in patients without prior CAD. Five (0.2% of patients) had a missing value for one of the Essen score variables and were excluded from subsequent analyses. Among patients without prior CAD, those with an Essen score ≤1 comprised 565 (28.1%) of all patients but accounted for 3 (4.3%) of follow-up MI, whereas those with a score ≥4 comprised 14.6% (n=294) of all patients but accounted for 31.4% (n=22) of MI (Table 3). There were also 413 recurrent ischemic strokes during follow-up, of which 313 (75.7%) occurred among patients without prior CAD.

**Table 3.**
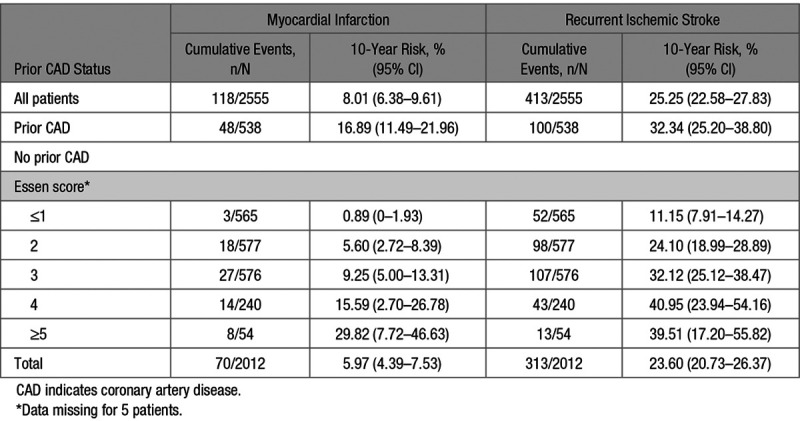
Ten-Year Risks of Myocardial Infarction and Recurrent Ischemic Stroke After Transient Ischemic Attack or Ischemic Stroke in Patients Without Prior CAD, Stratified by the Essen Risk Score, and in Those With Prior CAD

In patients without prior CAD, variables of the Essen score were all associated with an increased risk of MI after adjustment for age and sex (Table 4). The 10-year risk of MI in patients without prior CAD (overall risk =6.0% [95% CI, 4.4–7.5]) ranged from 0.9% (0–1.9) in those with an Essen score ≤1 to 29.8% (7.7–46.6) in those with a score ≥5 (Figure, Table 3), with a C statistic of 0.64 (0.57–0.71, *P*<0.001). Compared with patients with prior CAD (n=538, 21.1%), an Essen risk score of ≥4 (n=294, 11.5%) in those without prior CAD identified a subgroup at similarly high 10-year risk of MI (17.2%, 6.9–26.3; versus 16.9%, 11.5–22.0). The subgroup of patients without prior CAD with an Essen risk score of ≥4 were also at significantly higher 10-year risk of MI than those with a score of <3 (17.2%, 6.9–26.3 versus 4.7%, 3.2–6.2; *P*<0.001, age-and sex-adjusted hazard ratio =2.23, 1.32–3.76, Table 5, Table II in the online-only Data Supplement), with no interaction by TIA/stroke subtype (*P*interaction =0.93). The risk of MI was approximately linear with time in patients with prior CAD and in those without (Figure I in the online-only Data Supplement).

**Table 4.**
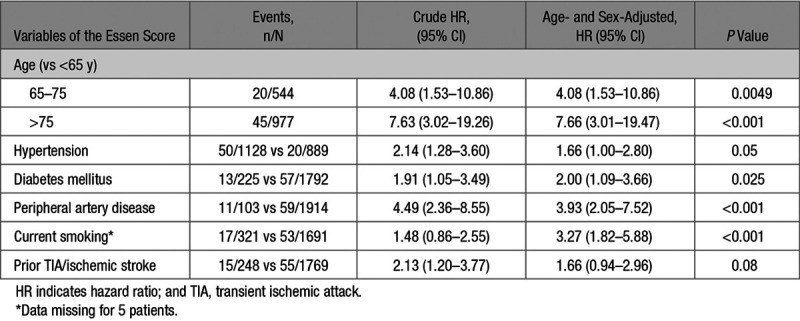
Variables of the Essen Score and Risk of Myocardial Infarction in Patients With TIA/Stroke Without Prior Coronary Artery Disease

**Table 5.**
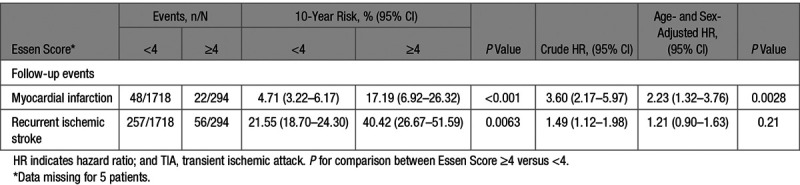
Stratification by Essen Score of ≥4 Versus <4 and Risk of Myocardial Infarction and of Recurrent Ischemic Stroke in Patients With TIA/Stroke Without Prior Coronary Artery Disease

**Figure.**
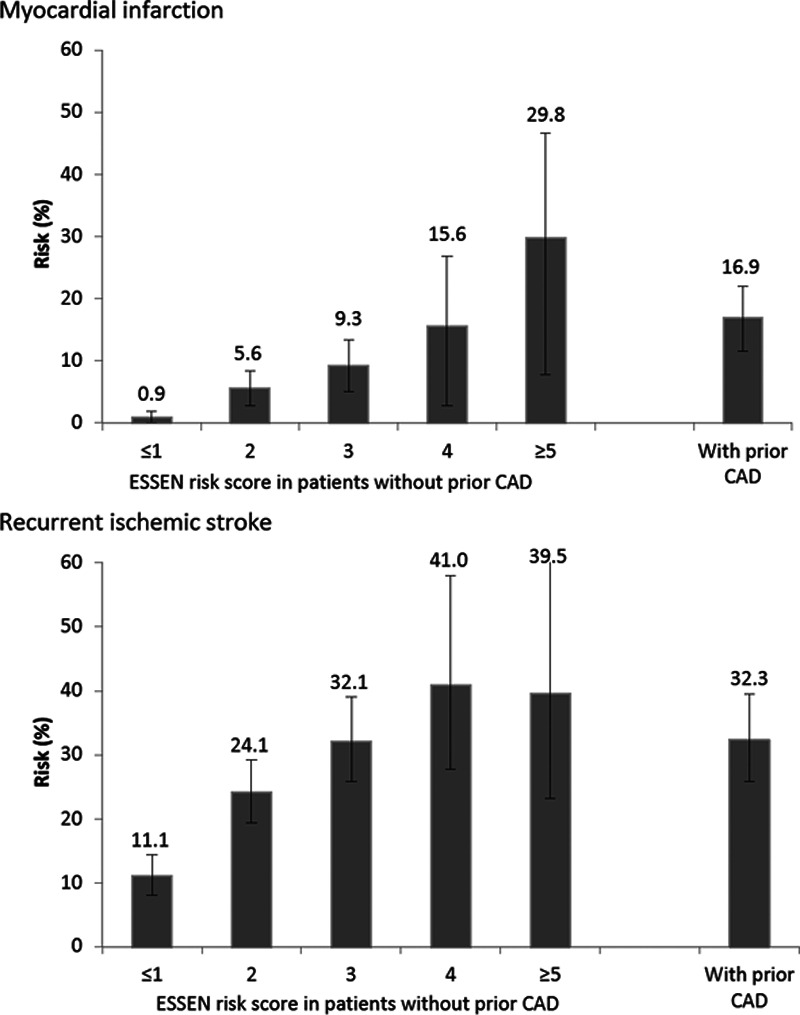
Ten-year actuarial risk of myocardial infarction and of recurrent ischemic stroke in transient ischemic attack/stroke patients based on the presence of prior coronary artery disease (CAD) and the Essen score.

The 10-year risk of recurrent ischemic stroke was 23.6% (20.7–26.4) overall in patients without prior CAD, ranging from 11.2% (7.9–14.3) in those with an Essen score ≤1 to 39.5% (17.2–55.8) in those with a score ≥5 (Figure, Table 3). The score tended to be less predictive (difference: *P*=0.0460) for the risk of recurrent ischemic stroke (C statistic =0.57, 0.54–0.60) than for the risk of MI.

An Essen risk score of ≥4 also identified a subgroup at similarly high 10-year risk of recurrent ischemic stroke to those with prior CAD (40.4%, 26.7–51.6; versus 32.4%, 25.2–38; Table 3). Although the risk of recurrent ischemic stroke increased with an increasing Essen score (Figure), the subgroup with an Essen risk score of ≥4 were not at significantly higher 10-year risk than those with an Essen risk score of <3 (40.4%, 26.7–51.6 versus 21.6%, 18.7–24.3, *P*=0.0063, age-and sex-adjusted hazard ratio =1.21, 0.90–1.63), with no interaction observed by TIA/stroke subtype (*P*-interaction=0.91, Table 5, Table II in the online-only Data Supplement). After the early high risk-period, the risk of recurrent ischemic stroke was approximately linear with time in patients with prior CAD and in those without (Figure II in the online-only Data Supplement).

In patients without prior CAD, 10-year risk of MI or recurrent ischemic stroke increased with an increasing Essen score in patients with large artery disease (TOAST) subtype and in those without (Table III in the online-only Data Supplement). Ten-year risk of MI or recurrent ischemic stroke in the subgroup of patients with large artery disease subtype (overall risk =27.5%, 2.37–34.1) ranged from 15.6% (5.1–25.0) in those with an Essen score ≤1 to 74.6% (16.2–92.3) in those with a score ≥5 while 10-year risk in the subgroup of patients with other TIA/stroke subtypes than large artery disease (overall risk =24.3%, 21.5–26.9) ranged from 10.5% (7.5–13.4) in those with an Essen score ≤1 to 50.0% (23.8–67.1) in those with a score ≥5.

Compared with cryptogenic subtype, cardioembolism, large artery disease, and small vessel disease subtypes were all associated with a higher risk of MI and of recurrent ischemic stroke after adjustment for age and sex, and the associations remained unchanged after further adjustment for the Essen score (Table IV in the online-only Data Supplement).

Adding the variable presence of large artery disease (TOAST) subtype as a categorical variable to the Essen score did not significantly increase the predictive value of the score for the risk of MI (C statistic =0.74, 0.57–0.71, Tables V and VI in the online-only Data Supplement) and of recurrent ischemic stroke (C statistic =0.58, 0.54–0.60).

Information on antithrombotic therapy at the time of the follow-up MI was available for 102 of the 118 patients with MI (87 on antiplatelet therapy only and 15 on anticoagulant therapy). Antithrombotic therapy had been withdrawn within 30 days of the MI in 7 (7%) patients (3 for bleeding and 4 for surgery, Table VII in the online-only Data Supplement). None of these 7 patients had prior CAD, but the MI was fatal in 5 cases.

## Discussion

In this population-based cohort of consecutive patients with TIA or ischemic stroke on current secondary prevention management, the Essen score risk-stratified individuals without prior CAD for the risk of follow-up MI and of recurrent ischemic stroke. An Essen score of ≥4 in those without prior CAD identified a subgroup at similarly high 10-year risk of MI to those with prior CAD and of recurrent ischemic stroke. Thus, in addition to the 538 patients with known prior CAD, an Essen risk score of ≥4 identified a further 257 patients with high-risk TIA/stroke. Since trials of more intensive secondary prevention have reported significant reductions in the risk of recurrent ischemic events in patients with prior CAD,^3–6^ trials of such treatment might also be justified in high-risk patients without prior CAD.

The Essen score was previously shown to predict the 1-year risk of recurrent ischemic stroke in patients with TIA/stroke due to large artery disease,^10,12–15^ but we have shown predictive value in a broader range of patients with longer follow-up. We also showed that the score predicted risk of MI better than recurrent stroke, perhaps because all patients already had established cerebrovascular disease, and so discrimination for stroke risk was more difficult. We found a 2-fold increase in the risk of MI and a 20% rise in the risk of recurrent ischemic stroke in patients with Essen score of ≥4 compared with those with a score of <4. We also found that the predictive value of the score was similar for patients with different TIA/stroke TOAST subtypes, although larger studies would be required to confirm this finding. In patients with TIA/stroke without prior CAD, a few scores have been shown to predict the presence of asymptomatic coronary lesions (PRECORIS score [Predicting Asymptomatic Coronary Artery Disease in Patients With Ischemic Stroke and Transient Ischemic Attack])^25,26^ or the risk of MI (Framingham score).^27^ However, these scores involved patients recruited in trials or hospital-based cohorts, with more potential selective biases than population-based cohorts, and only looked at the short-term risk and their clinical utility for predicting the long-term risk is unknown. A high Essen risk score in patients with TIA/stroke without prior CAD identified subgroups at substantially high risk of follow-up MI. However, whether these individuals should be screened for asymptomatic coronary lesions is unclear.

No study has assessed whether coronary revascularisation in patients with TIA/stroke screened for asymptomatic CAD might reduce future cardiac events.

Although we consider our findings to be valid, our study has limitations. First, our cohort was predominantly made up of white patients, which might limit generalisability. Second, although we aimed for very high rates of secondary prevention, our population-based cohort included many frail and very elderly patients, often with dementia, prior bleeding, or terminal illness, and some patients refused medication, or it was contraindicated for other reasons. However, 1-year medication rates were higher than in other similar cohorts,^28–31^ and can be regarded as being based closely on current guidelines for secondary prevention. We also showed that very few MIs were accounted for by discontinuation of antithrombotic therapy. Third, in our cohort, baseline characteristics were well-defined, and although we make recommendations to primary care physicians about changes to risk factors and medication at study follow-up visits, we have limited information on the control of vascular risk factors during the whole follow-up. Fourth, we excluded the other cardiovascular disease variable from our analysis of the Essen score due to a lack of clarity of what conditions and what severity of disease are required to satisfy the definition.^10^ However, this omission is likely to have reduced predictive value, and so our findings are probably somewhat conservative. The C statistics of the score were of moderate predictive value for the risk of MI and of recurrent ischemic stroke, however, in certain clinical situations, the score would still provide clinically important information. In the subgroups of patients with TIA/stroke without prior CAD and with a score of ≥4, 10-year risk of MI was 17%, and 10-year risk of recurrent ischemic stroke was 40%, which was as high as in patients with prior CAD.

In conclusion, the Essen score is a simple clinical score to risk-stratify patients with TIA/stroke without prior CAD, and to identify patients who may be at sufficiently high risk of MI and recurrent stroke to justify more intensive treatment or inclusion in trials.

## Acknowledgments

We are grateful to all the staff in the general practices that collaborated in OXVASC (Oxford Vascular Study). We also acknowledge the use of the facilities of the Acute Vascular Imaging Centre, Oxford. The views expressed are those of the author(s) and not necessarily those of the National Health Service, the National Institute for Health Research, or the Department of Health.

## Sources of Funding

This work was supported by National Institute for Health Research (NIHR) Oxford Biomedical Research Centre, Wellcome Trust, Wolfson Foundation and the British Heart Foundation. P.M. Rothwell is in receipt of a NIHR Senior Investigator award. MB was funded by Société Française de Neurovasculaire (SFNV)-France AVC, Journées de Neurologie en Langue Française (JNLF), Fondation Planiol pour l’Etude du Cerveau & Institut Servier. Ethics approval: The Oxford Vascular Study was approved by the Oxfordshire clinical research ethics committee (CO.043).

## Disclosures

None.

## Supplementary Material
