# Supplementary material for: Essen Risk Score in Prediction of Myocardial Infarction After Transient Ischemic Attack or Ischemic Stroke Without Prior Coronary Artery Disease
Source: Stroke. 2019 Oct 22;50(12):3393–9. doi: 10.1161/STROKEAHA.119.025831 (PMC7597993; doi:10.1161/STROKEAHA.119.025831)

Essen risk score in prediction of myocardial infarction after TIA or ischemic stroke without prior coronary artery disease

SUPPLEMENTAL MATERIAL

Supplemental Table I: Criteria for the modified TOAST classification for stroke

Supplemental Table II: Stratification by Essen score of ≥4 versus <4 and risk of myocardial infarction and of recurrent ischemic stroke in TIA/stroke patients without prior coronary artery disease, stratified by TIA/stroke subtype (TOAST classification)

Supplemental Table III: 10-year risk of myocardial infarction or recurrent ischemic stroke after TIA or ischemic stroke based on presence of large artery disease (TOAST) subtype in patients without prior coronary artery disease (CAD), stratified by the Essen risk score, and in those with prior CAD

Supplemental Table IV: Association between TIA/stroke subtype (TOAST classification) and risk of myocardial infarction and of recurrent ischemic stroke in TIA or recurrent ischemic stroke patients without prior coronary artery disease

Supplemental Table V: 10-year risk of myocardial infarction and of recurrent ischemic stroke after TIA or ischemic stroke in patients without prior coronary artery disease (CAD), stratified by the modified Essen risk score (after adding the variable “presence of large artery disease TIA/Stroke subtype” to the score), and in those with prior CAD

Supplemental Table VI: Stratification by modified Essen score (after adding the variable “presence of large artery disease subtype” to the score) of ≥4 versus <4 and risk of myocardial infarction and of recurrent ischemic stroke in TIA/stroke patients without prior coronary artery disease

Supplemental Table VII: Characteristics of the seven TIA or ischemic stroke patients who experienced a myocardial infarction (MI) during follow-up after withdrawal of antithrombotic therapy

Supplemental Figure I: Risk of follow-up myocardial infarction in TIA or ischemic stroke patients stratified by presence of prior coronary artery disease (CAD)

Supplemental Figure II: Risk of recurrent ischemic strokes in TIA or ischemic stroke patients stratified by presence of prior coronary artery disease (CAD)

Supplemental Table I: Criteria for the modified TOAST classification for stroke etiology[1]

TOAST subtypes	Definition
Large artery disease (LAD)	Imaging abnormality of atherosclerosis of intra or extracranial artery supplying the ischemic filed with either occlusive or stenosis (≥50% diameter reduction).
Small vessel disease (SVD)	Clinical lacunar syndromes with no cerebral cortical dysfunction and normal imaging; OR<br>Imaging evidence of a relevant acute infarction <20 mm within the territory of basal or brainstem penetrating arteries in the absence of any other pathology in the parent artery at the site of the origin of the penetrating artery.
Cardioembolic (CE)	Major-risk cardioembolic source of embolism<br>• Prosthetic cardiac valve<br>• Mitral stenosis<br>• Permanent or paroxysmal AF (>30s)<br>• Sustained atrial flutter<br>• Intracardiac thrombus<br>• Sick sinus syndrome<br>• Recent myocardial infarction (<4 weeks)<br>• Atrial myxoma or other cardiac tumors<br>• Infective endocarditis<br>• Valvular vegetations<br>• Congestive heart failure (Ejection fraction <30%)<br>• PFO and concomitant PE or DVT or long-haul flight preceding the cerebrovascular event with clinical presentation of an embolic event
Unknown causes	The absence of diagnostic tests that, under the examiner's judgment, their presence would have been essential to uncover the underlying etiology.
Other determined causes	The presence of a specific disease process that involves clinically appropriate brain arteries and disorders that bear a clear and close temporal or spatial relationship with the acute event.<br>• Abnormalities od thrombosis and hemostasis<br>• Arterial dissection<br>• Acute disseminated intravascular coagulation<br>• Clinically relevant aneurysm<br>• Drug-induced event<br>• Fibromuscular dysplasia<br>• Hyperviscosity syndromes<br>• Hypoperfusion syndromes<br>• Iatrogenic causes<br>• Meningitis<br>• Migraine-induced event<br>• Mitochondrial myopathy encephalopathy with lactic acidosis and stroke-like episodes<br>• Moyamoya disease<br>• Primary antiphospholipid antibody syndrome<br>• Primary infection of the arterial wall<br>• Segmental vasoconstriction or vasospasm<br>• Sickle cell disease<br>• Sneddon syndrome<br>• Thrombotic thrombocytopenic purpura<br>• Other
Several causes	The presence of >1 evident mechanism in which there is either probable evidence for each.
Cryptogenic	Patient who has had at least one type of brain imaging (CT/MR/autopsy), ECG and at least one type of vascular imaging (Carotid Doppler/MRA/CTA/DSA/TCD/autopsy) but no etiology was identified.

1. Adams HP, Jr., Bendixen BH, Kappelle LJ, Biller J, Love BB, Gordon DL, et al. Classification of subtype of acute ischemic stroke. Definitions for use in a multicenter clinical trial. Toast. Trial of org 10172 in acute stroke treatment. *Stroke*. 1993;24:35-41

Supplemental Table II: Stratification by Essen score of ≥4 versus <4 and risk of myocardial infarction and of recurrent ischemic stroke in TIA/stroke patients without prior coronary artery disease, stratified by TIA/stroke subtype (TOAST classification)

	**10-year risk, % (95% CI)**		**p**	**Crude Hazard Ratio (95%CI)**	**Age- and sex-adjusted Hazard Ratio (95%CI)**	**p**
**Essen score***	<4	≥4				
**All patients**						
Myocardial infarction	4.71 (3.22-6.17)	17.19 (6.92-26.32)	<0.001	3.60 (2.17-5.97)	2.23 (1.32-3.76)	0.0028
Recurrent ischemic stroke	21.55 (18.70-24.30)	40.42 (26.67-51.59)	0.0063	1.49 (1.12-1.98)	1.21 (0.90-1.63)	0.21
**Stroke subtypes**				*p*-interaction=0.93 for myocardial infarction<br>*p*-interaction=0.91 for recurrent ischemic stroke		
**Cardioembolic**						
Myocardial infarction	7.10 (1.88-12.04)	8.23 (0-18.60)	0.45	1.61 (0.45-5.73)	1.09 (0.30-3.98)	0.90
Recurrent ischemic stroke	29.10 (20.26-36.97)	35.65 (13.31-52.23)	0.22	1.42 (0.81-2.50)	1.07 (0.60-1.89)	0.81
**Large artery disease**						
Myocardial infarction	7.39 (1.90-12.57)	28.56 (10.29-43.10)	<0.001	7.55 (2.87-19.86)	5.69 (1.81-17.88)	0.0029
Recurrent ischemic stroke	21.61 (12.81-29.52)	47.97 (22.74-64.96)	0.0014	2.74 (1.44-5.23)	2.90 (1.40-60.2)	0.0043
**Small vessel disease**						
Myocardial infarction	4.36 (1.24-7.39)	19.72 (0-35.91)	0.0014	5.72 (1.71-19.19)	3.57 (0.92-13.92)	0.06
Recurrent ischemic stroke	22.18 (15.43-28.39)	51.73 (0-79.95)	0.93	0.96 (0.38-2.41)	1.01 (0.38-2.66)	0.90
**Cryptogenic**						
Myocardial infarction	2.78 (0.92-4.61)	11.10 (0-24.14)	0.17	2.38 (0.66-8.54)	1.16 (0.32-4.20)	0.82
Recurrent ischemic stroke	15.40 (11.78-18.87)	37.51 (15.38-53.86)	0.0440	1.73 (1.01-2.97)	1.31 (0.74-2.29)	0.35

*Data on Essen score missing in 5 patients. *p*-interaction between the TIA/stroke subtypes

Supplemental Table III: 10-year risk of myocardial infarction or recurrent ischemic stroke after TIA or ischemic stroke based on presence of large artery disease (TOAST) subtype in patients without prior coronary artery disease (CAD), stratified by the Essen risk score, and in those with prior CAD

**Prior CAD status**	**Large artery disease TIA/stroke subtype**		**All other TIA/stroke subtypes**	
	**Cumulative events, n/N**	**10-year risk, % (95% CI)**	**Cumulative events, n/N**	**10-year risk, % (95% CI)**
**No prior CAD**				
Essen score*:				
≤1	8/52	15.62 (5.06-25.00)	47/513	10.48 (7.45-13.42)
2	13/62	25.20 (11.50-36.79)	97/514	24.91 (19.85-29.65)
3	11/62	23.46 (8.68-35.86)	116/514	35.50 (28.62-41.72)
4	13/31	48.09 (22.66-65.16)	39/209	41.60 (24.71-54.70)
≥5	6/9	74.60 (16.20-92.30)	14/45	49.97 (23.80-67.15)
Total	51/216	27.54 (20.37-34.08)	313/1795	24.27 (21.52-26.94)
**Prior CAD**	19/64	39.50 (22.41-52.82)	116/473	38.25 (31.28-44.50)

CI: confidence interval, *data missing for 5 patients
All other TIA/stroke subtypes include cardioembolism, small vessel disease, cryptogenic, more than one cause, unknown and other causes

Supplemental Table IV: Association between TIA/stroke subtype (TOAST classification) and risk of myocardial infarction and of recurrent ischemic stroke in TIA or recurrent ischemic stroke patients without prior coronary artery disease

TIA/stroke subtype	Crude Hazard Ratio (95%CI)	Age-and sex-adjusted Hazard Ratio (95%CI)	Age-, sex- and Essen score-adjusted Hazard Ratio (95%CI)	p
**Cardioembolic**				
Myocardial infarction	2.52 (1.21-5.24)	1.95 (0.93-4.08)	1.97 (0.94-4.13)	0.07
Recurrent ischemic stroke	1.97 (0.94-4.13	1.64 (1.20-2.23)	1.60 (1.18-2.18)	0.0028
**Large artery disease**				
Myocardial infarction	4.37 (2.15-8.87)	4.07 (2.00-8.29)	3.58 (1.76-7.30)	<0.001
Recurrent ischemic stroke	3.58 (1.76-7.30)	1.66 (1.15-2.40)	1.55 (1.08-2.24)	0.0186
**Small vessel disease**				
Myocardial infarction	2.11 (0.98-5.46)	2.19 (1.01-4.75)	2.13 (1.00-4.61)	0.05
Recurrent ischemic stroke	2.13 (0.98-4.61)	1.43 (1.01-2.02)	1.38 (0.98-1.95)	0.06
**Cryptogenic**	1 (reference)	1 (reference)	1 (reference)	-

CI: confidence interval

Supplemental Table V: 10-year risk of myocardial infarction and of recurrent ischemic stroke after TIA or ischemic stroke in patients without prior coronary artery disease (CAD), stratified by the modified Essen risk score (after adding the variable "presence of large artery disease TIA/Stroke subtype" to the score), and in those with prior CAD

Prior CAD status	Myocardial infarction		Recurrent ischemic stroke	
	Cumulative events, n/N	10-year risk, % (95% CI)	Cumulative events, n/N	10-year risk, % (95% CI)
**No prior CAD**				
Modified Essen score*:				
≤1	3/522	0.96 (0-2.08)	44/522	10.46 (7.12-13.68)
2	14/557	4.45 (1.77-7.06)	95/557	23.58 (18.55-28.31)
3	27/576	9.16 (5.12-13.04)	109/576	32.18 (25.24-38.47)
4	13/271	12.75 (3.41-21.19)	40/271	33.19 (20.26-44.02)
≥5	13/86	24.62 (10.74-36.34)	25/86	45.04 (27.02-58.62)
Total	70/2012	5.97 (4.39-7.53)	313/2012	23.60 (20.73-26.37)
**Prior CAD**	48/538	16.89 (11.49-21.96)	100/538	32.34 (25.20-38.80)

CI: confidence interval, *data missing for 5 patients.
Modified Essen score in patients without prior CAD ranged from 0 to 8: 1 point is attributed for age 65-75 years; 2 for age >75 years and 1 for each history of prior hypertension, diabetes mellitus, peripheral artery disease, current smoking or previous TIA or ischemic stroke; and 1 point for presence of large artery disease (TOAST) subtype.

In TIA/stroke patients without prior CAD, the c-statistic score of the modified Essen score was 0.74 (95%CI 0.68-0.79) for the risk of myocardial infarction and 0.58 (95%CI 0.54-0.60) for the risk of recurrent ischemic stroke.

Supplemental Table VI: Stratification by modified Essen score (after adding the variable "presence of large artery disease subtype" to the score) of ≥4 versus <4 and risk of myocardial infarction and of recurrent ischemic stroke in TIA/stroke patients without prior coronary artery disease

	**Events, n/N**		**10-year risk, % (95% CI)**		**p**	**Crude HR (95%CI)**	**Age- and sex-adjusted HR (95%CI)**	**p**
**Modified Essen score***	<4	≥4	<4	≥4				
Follow-up events:								
Myocardial infarction	44/1655	26/357	4.46 (2.99-5.90)	15.67 (7.75-22.92)	<0.001	3.49 (2.15-5.68)	2.21 (1.33-3.65)	0.0020
Recurrent ischemic stroke	248/1655	65/357	21.50 (18.60-24.29)	36.37 (25.59-45.60)	0.0146	1.40 (1.07-1.83)	1.44 (0.87-1.51)	0.34

CI: confidence interval, *data missing for 5 patients. HR: hazard ratio, p for comparison between modified Essen score ≥4 versus <4.
Modified Essen scores in patients without prior CAD ranged from 0 to 8: 1 point is attributed for age 65-75 years; 2 for age >75 years and 1 for each history of prior hypertension, diabetes mellitus, peripheral artery disease, current smoking or previous TIA or ischaemic stroke; and 1 point for presence of large artery disease (TOAST) subtype.

Supplemental Table VII: Characteristics of the seven TIA or ischemic stroke patients who experienced a myocardial infarction (MI) during follow-up after withdrawal of antithrombotic therapy

Sex	Age (years)	CAD	PAD	HTN	DM	Previous TIA/IS	Current smoking	Essen score	TIA/stroke subtype	Antithrombotic drug used prior to the MI	Reason for withdrawal of antithrombotic drug	Date (type) of stroke	Date (type) of MI	Date (cause) of death
F	71	No	No	Yes	No	Yes	No	3	CE	Aspirin	Surgery	10/08/02 (TIA)	12/08/02 (N-STEMI)	No
F	92	No	No	Yes	Yes	Yes	No	5	SVD	Aspirin	Bleeding	27/08/02 (IS)	09/03/05 (STEMI)	09/03/05 (cardiac)
F	84	No	Yes	Yes	No	Yes	Yes	6	LAD	Clopidogrel	Bleeding	04/03/03 (IS)	23/05/03 (N-STEMI)	06/06/03 (non-cardiac)
F	88	No	No	Yes	No	No	No	3	Crypt.	Aspirin + Clopidogrel	Surgery	08/09/03 (TIA)	02/10/03 (N-STEMI)	21/12/05 (cardiac)
M	91	No	No	Yes	No	Yes	No	4	Crypt.	Clopidogrel	Surgery	17/08/05 (TIA)	30/10/07 (N-STEMI)	30/10/07 (cardiac)
M	92	No	No	No	No	Yes	No	3	Crypt.	Aspirin	Surgery	25/12/05 (TIA)	28/10/08 (N-STEMI)	10/11/08 (cardiac)
M	68	No	No	Yes	No	No	No	2	SVD	Rivaroxaban	Bleeding	11/10/10 (IS)	25/09/14 (STEMI)	No

Discontinuation was recommended by a physician-based for 5 patients (2 for surgery and 3 for bleeding) and the reason was not documented for the other 2 patients M: male, F: female, CAD: coronary artery disease, PAD: peripheral artery disease, HTN: hypertension, DM: diabetes mellitus, IS: ischemic stroke, CE: cardioembolic, LAD: large artery disease, SVD: small vessel disease, Crypt.: cryptogenic, STEMI: ST-elevation myocardial infarction, N-STEMI: Non-ST-elevation myocardial infarction, cardiac: cardiac death, non-cardiac: death not attributable to cardiac cause

Supplemental Figure I: Risk of follow-up myocardial infarction in TIA or ischemic stroke patients stratified by history of presence of prior coronary artery disease

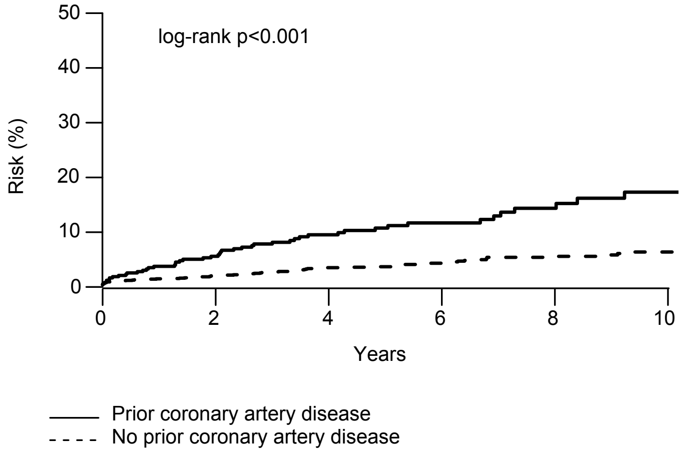

Supplemental Figure II: Risk of recurrent ischemic strokes in TIA or ischemic stroke patients stratified by presence of prior coronary artery disease

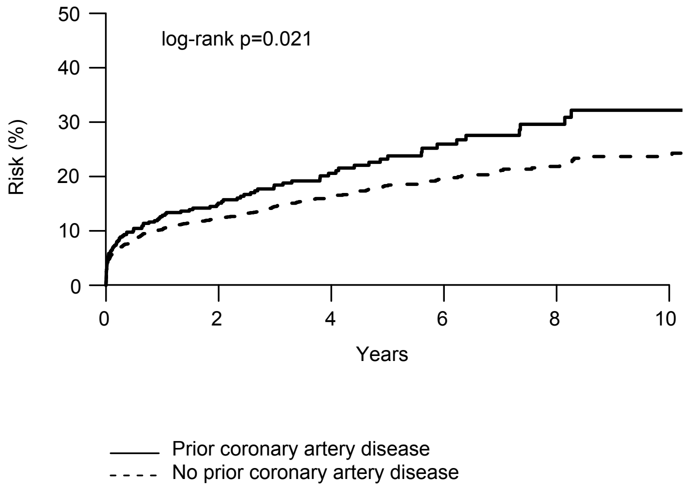

Supplement: Supplementary file 1 [file str-50-3393-s001.pdf]
